# Pain, opioid consumption, and epidural anesthesia in patients with inflammatory bowel disease undergoing laparoscopic subtotal colectomy: an observational cohort study

**DOI:** 10.1007/s10151-025-03118-2

**Published:** 2025-03-07

**Authors:** C. Ryrsø, T. Fransgård, L. P. K. Andersen

**Affiliations:** 1 Department of Anesthesia, Zealand University Hospital, Lykkebækvej 1, 4600 Køge, Denmark; 2 Department of Surgery, Zealand University Hospital, Køge, Denmark; 3 Department of Clinical Medicine, University of Copenhagen, Copenhagen, Denmark; 4 Center for Surgical Science, Zealand University Hospital, Køge, Denmark

**Keywords:** Inflammatory bowel disease, Colectomy, Pain, Opioid, Epidural anesthesia

## Abstract

**Background:**

Surgery is often needed to provide disease control in patients with inflammatory bowel disease. Studies document increased postoperative pain and complicated perioperative courses. This study examines postoperative pain and opioid consumption in patients with inflammatory bowel disease undergoing laparoscopic subtotal colectomy. Furthermore, the impact of epidural anesthesia is investigated.

**Methods:**

This study encompassed an observational cohort of patients with inflammatory bowel disease undergoing subtotal colectomy in the period 1 January 2018 to 30 June 2023 at a university hospital in Denmark. Demographic and perioperative data, opioid consumption, pain scores, and procedural data of epidural anesthesia were retrieved from patient records. Data were stratified according to the use of epidural anesthesia.

**Results:**

The study included 153 patients. Overall, 45% of patients received epidural anesthesia. Opioid consumption in the postoperative care unit was 9.2 mg (3.3–15.8 mg) and 3.8 mg (0–15 mg) (*P* = 0.04) in patients without and with epidural anesthesia, respectively. Correspondingly, opioid consumption during the first 24 h postoperatively was 23.3 mg (10–33 mg) and 6.8 mg (0–21.7 mg) (*P* < 0.001). Numerical rating scale (NRS) pain in the postoperative care unit was 3.5 (2–4.6) and 2.7 (1.3–4.3) in patients without and with epidural anesthesia, respectively (*P* = 0.1645). Thirty percent of patients treated with epidural anesthesia experienced ≥ 1 adverse event(s) related to epidural anesthesia.

**Conclusions:**

Our study demonstrates a relatively low consumption of opioids and low pain scores in the early postoperative period following laparoscopic subtotal colectomy regardless of the use of epidural anesthesia. Epidural anesthesia was associated with a substantial frequency of adverse events.

## Introduction

The prevalence of inflammatory bowel disease (IBD) in Denmark is 295/100,000 person-years and 589/100,000 person-years for Crohn’s disease (CD) and ulcerative colitis (UC), respectively [1]. Medical treatments include aminosalicylates, corticosteroids, and biological agents [2, 3]. Within 5 years of diagnosis, 5–10% of patients with UC and 10–30% of patients with CD will undergo surgery to achieve disease control [4]. The surgical procedures differ according to the individual patient and affected gastrointestinal tract segment [2, 5]. Owing to the chronic and unpredictable nature of IBD, surgery may be performed in both an acute-, subacute-, or elective setting.

Implementation of enhanced recovery after surgery (ERAS) programs in cancer surgery has improved perioperative outcomes [6]. In contrast, surgery in patients with IBD still demonstrates increased lengths of stay and increased risks of postoperative complications, irrespective of ERAS [7]. Optimal analgesia is pivotal in any surgical patient. However, the pathophysiology of IBD, including previous and potentially ongoing inflammation, seems to alter the patients’ perception of pain [8, 9] and weaken treatment response to conventional analgesic treatments [10]. Moreover, certain well-established analgesics, such as non-steroidal anti-inflammatory drugs (NSAIDs), are contraindicated in these patients [11]. So far, no clinical guidelines have defined an optimal perioperative analgesic regimen for patients with IBD undergoing colectomy.

The aim of this study is to investigate opioid consumption and postoperative pain in patients with IBD undergoing subtotal colectomy with minimal invasive surgery (MIS) technique. Furthermore, to describe perioperative outcomes in patients with or without epidural anesthesia. Finally, to provide data concerning the clinical impact of epidural anesthesia, including the occurrence of adverse events.

## Methods

### Study design

The study is an observational cohort study. The cohort included consecutive patients with IBD undergoing subtotal colectomy at Zealand University Hospital (ZUH), Denmark in the period 1 January 2018 to 30 June 2023. Data were retrieved from the electronic patient records, and included demographic and perioperative data, opioid consumption, pain scores, and clinical procedural data of epidural anesthesia.

### Perioperative course

All surgical procedures were planned as MIS and performed by dedicated colorectal surgeons. The primary surgical procedure was subtotal colectomy including ileostomy. Pouch surgery was not performed at ZUH. General anesthesia included total intravenous anesthesia (TIVA) with propofol and remifentanil. Neuromuscular blockade was employed at the discretion of the anesthesiologist/surgeon. Infiltration of local anesthetics, 40 mL bupivacaine 2.5 mg/mL, was employed at port sites. Intravenous morphine 0.2 mg/kg was administered 30 min before end of surgery. PONV was treated with dexamethasone 4 mg. In the post-anesthesia care unit (PACU), morphine or oxycodone was administered as needed, assessed by the PACU staff. Pain in the PACU was evaluated by the treating nurse employing the numerical rating scale (NRS). All patients were assessed at a minimum upon arrival and departure from the PACU. Perioperative epidural anesthesia and other types of nerve blockades were placed at the discretion of the anesthesiologist. The postoperative analgesic regimen in the ward consisted of paracetamol 1 g × 4 and morphine 10 mg orally or 5 mg intravenously (IV), as needed. Perioperative care adhered to the local ERAS protocol, including MIS, avoidance of drains/catheters, early enteral nutrition, and mobilization. ERAS was initiated from July 2020 onward.

### Statistics

Stratification of patient data was performed on the basis of the use of epidural anesthesia in the perioperative course—referred to as the “non-epi group” and the “epi group.” The use of epidural anesthesia was defined as the placement of an epidural catheter in relation to (before or immediately after) the primary subtotal colectomy. All reported data refer to these procedures. Primary outcomes were opioid consumption in the PACU and during the first 24 h postoperatively. Secondary outcomes included NRS in the PACU and adverse events related to epidural anesthesia. Consumption of opioids is reported as intravenous morphine equivalents (IME). Other types of opioids administered during the perioperative course are converted accordingly [12]. Normality of data was assessed by visual inspection of the distribution of data. Nonparametric statistics was applied accordingly. Data are reported as median (interquartile range) and percentages. Comparisons between the groups were performed using the Mann–Whitney test. A *P*-value below 0.05 was considered statistically significant. Data was analyzed using R (R-4.3.2 for Windows, Posit Software, PBC). Relevant approvals according to Danish law were provided by the Danish Data Protection Agency (REG-093–2020) and the Danish Patient Safety Authority (STPS: 31–1521-451).

## Results

A total of 153 patients were included in the study. Patient demographics are presented in Table 1. Preoperative opioid use was demonstrated in 10% and 7% of patients, respectively. In the non-epi group and epi group, 57% and 52%, respectively, suffered from one or more comorbidities. Perioperative outcomes are presented in Table 2. Opioid consumption in the PACU was 9.2 mg IME (3.3–15.8 mg) and 3.8 mg IME (0–15 mg) and differed significantly between the groups (*P* = 0.04). Correspondingly, opioid consumption during the first 24 h postoperatively was 23.3 mg IME (10–33 mg) and 6.8 mg IME (0–21.7 mg) (*P* < 0.001). NRS in the PACU was 3.5 (2–4.6) and 2.7 (1.3–4.3) in patients without and with epidural anesthesia, respectively (*P* = 0.1645). Length of stay (LOS) was 4.5 days (4–7 days) and 7 days (6–12 days). Occurrence of reoperation within 30 days postoperatively were 18% in the non-epi and 12% in the epi group.

**Table 1:** Patient demographics

	No epidural group (*N* = 84)		Epidural group (*N* = 69)	
	*n* (%)	Median (IQR)	*n* (%)	Median (IQR)
Number of patients	84 (55)		69 (45)	
Age (years)		53 (32–64.5)		47 (30–63)
Gender				
Male	40 (48)		42 (61)	
Female	44 (52)		27 (39)	
BMI (kg/m^2^)		24.1 (21.2–27.8)		23.7 (20.9–28.9)
ASA-score		2 (2–2)		2 (2–2)
IBD subtype				
Crohn’s disease	16 (19)		23 (33)	
Ulcerative colitis	67 (80)		42 (61)	
Undetermined IBD	1 (1)		4 (6)	
Preoperative opioid consumption	8 (10)		5 (7)	
Comorbidity				
Cardiovascular	26 (31)		13 (19)	
Pulmonary	13 (15)		9 (13)	
Endocrine	13 (15)		8 (12)	
Malignancy	2 (2)		2 (3)	
Psychiatric	8 (10)		8 (12)	
Liver disease	2 (2)		2 (3)	
Kidney disease	1 (1)		1 (1)	
Other	10 (12)		17 (25)	

*IQR* interquartile range, *BMI* body mass index, *IBD* inflammatory bowel disease

ASA-score: The American Society of Anesthesiologists physical status classification system (1–6)

Cardiovascular: hypertension, ischemic heart disease, heart failure, and stroke or acute myocardial infarction

Pulmonary: chronic obstructive pulmonary disease, asthma, and pulmonary fibrosis

Endocrine: diabetes mellitus type 1 and 2, obesity, hypo- and hyperthyroidism

Malignancy: solid or hematological cancer

Psychiatric: depression, bipolar disorder, anxiety disorder, and psychotic disorders

Liver disease: liver cirrhosis, hepatitis, liver failure, primary sclerosing cholangitis, and primary biliary cholangitis

Kidney disease: chronic kidney disease stage 1–5 as defined by Kidney Disease: Improving Global Outcomes

**Table 2:** Perioperative outcomes

	No epidural group (*N* = 84)		Epidural group (*N* = 69)		*P*-value
	*n* (%)	Median (IQR)	*n* (%)	Median (IQR)	
Minimal invasive surgery	83 (99)		65 (94)		
Converted from laparoscopic to open surgery	0 (0)		7 (11)		
Surgery priority					
Elective	37 (44)		28 (41)		
Subacute	44 (52)		33 (48)		
Acute	3 (4)		8 (12)		
LOS in the surgical ward		4.5 (4–7)		7 (6–12)	
Opioid consumption in the PACU^a^		9.2 (3.3–15.8)		3.8 (0–15)	0.04*
Opioid consumption 24 h postoperatively^a^		23.3 (10–33)		6.8 (0–21.7)	< 0.001*
NRS score in the PACU		3.5 (2–4.6)		2.7 (1.3–4.3)	0.16
Patients receiving additional nerve blockades^b^	15 (18)		7 (10)		
Patients assessed by the acute pain service unit	12 (14)		35 (51)		
In hospital perioperative adverse events					
ICU	1 (1)		4 (6)		
Death	1 (1)		1 (1)		
Reoperation	8 (10)		8 (12)		
Reoperation in total^c^	15 (18)		8 (12)		

*LOS* length of stay, *PACU* post-anesthesia care unit, *NRS* numerical rating scale, *ICU* intensive care unit

*P-value < 0.05

^a^IME (intravenous morphine equivalent) dose in mg

^b^Not epidural anesthesia

^c^Includes patients who had reoperation within 30 days postoperatively

Characteristics of epidural anesthesia are presented in Table 3. Overall, 45% percent of patients were treated with epidural anesthesia, of which 58% were placed preoperatively, while the remaining 42% were placed within 24 h after surgery. In patients treated with epidural anesthesia, lack of/or insufficient effect requiring intervention was observed in 18% of patients. Thirty percent of patients treated with epidural anesthesia experienced ≥ 1 adverse events related to epidural anesthesia.

**Table 3:** Characteristics of epidural anesthesia

	Epidural	
	*n* (%)	Median (IQR)
Placed preoperatively	46 (58)	
Placed within 24 h postoperatively	33 (42)	
Placed by an anesthesia specialist	46 (70)	
Thoracic level		
Th 7/8	3 (4)	
Th 8/9	19 (25)	
Th 9/10	33 (43)	
Th 10/11	19 (25)	
Th 11/12	3 (4)	
Difficult placement^d^	6 (8)	
Number of POD with epidural anesthesia		3 (2–4)
Patients experiencing lack of/insufficient effect	14 (18)	
Adverse events		
Patients with ≥ 1 adverse events	21 (30)	
Paresis and/or paresthesia in the lower extremities	8 (10)	
Dizziness	6 (8)	
Hypotension	4 (5)	
Urinary retention	3 (4)	
Pruritus	1 (1)	
PDPH	1 (1)	
Failed epidural blockade placement	2	

*POD* postoperative days, *PDPH* post-dural puncture headache

^d^Difficult placement was defined as ≥ three attempts or need of assistance from a colleague

## Discussion

In this observational cohort study, we found a relatively low consumption of opioids in the PACU, and 24 h postoperatively, regardless of the use of epidural anesthesia in patients with IBD undergoing MIS subtotal colectomy. There was, however, a significant difference between the two groups regarding opioid consumption, both in the PACU and 24 h postoperatively. Correspondingly, pain scores in the early postoperative period were acceptably low. Despite MIS and an ERAS setting, LOS and reoperation rates were substantial. In patients treated with epidural anesthesia, clinical interventions were often needed to improve the analgesic effect. Also, adverse events related to epidural anesthesia occurred frequently.

ERAS is the backbone of modern surgical treatment. It includes a number of components, such as preoperative patient education, a short fasting period, MIS, restricted intraoperative fluid therapy, early mobilization, and enteral nutrition [13]. ERAS has been shown to improve postoperative outcomes within various surgical procedures [14–17]. ERAS also highlights the importance of optimal analgesic treatment [18]. Traditionally epidural anesthesia has been considered the gold standard in major colorectal surgery [19]. In open procedures, epidural anesthesia reduces pain and opioid consumption, improves lung function, and accelerates return of bowel function [20, 21]. In MIS, these advantages are not clear [22, 23], and consequently, epidural anesthesia is not recommended in elective colorectal cancer surgery [24]. To date, no randomized studies have examined epidural anesthesia in MIS colectomy in patients with IBD, and no clinical guidelines have defined an optimal analgesic regimen. Still, patients with IBD constitute a major clinical challenge in the perioperative course [25]. Our data underline this issue, demonstrating extended LOS and significant reoperation rates. Our data are supported by a previous Danish study documenting a LOS of 6 days for laparoscopic colorectal IBD surgery compared with 3 days in laparoscopic colorectal cancer surgery [26, 27]. Though our data demonstrate acceptable levels of pain and opioid consumption [28–31], a number of previous studies indicate suboptimal perioperative analgesia in patients with IBD [32–34]. It is hypothesized that these differences result from a generally younger, and primarily female patient population [35]. An additional cause may be pre-existing chronic pain states. A significant number of patients with IBD report pain as a chronic symptom [10, 36, 37]. Also, patients with IBD demonstrate an increased opioid consumption both in-hospital and as outpatients [37–39]. It is speculated that the effect of chronic inflammation may alter nociception, leading to central sensitization and increased clinical pain [9]. Finally, pain management in patients with IBD is challenged by the fact that effective analgesics, such as NSAIDs, are often avoided owing to the risk of enteropathy and gastropathy [11]. In conclusion, patients with IBD constitute a fragile patient population with an increased risk of complicated surgical courses. Further high-quality randomized trials investigating perioperative analgesic interventions to improve outcomes in this patient group are warranted. Studies should aim at exploring the optimal use of epidural anesthesia in either selected patients with IBD (e.g., multi-morbid, obese, or chronic pain) or in patients with IBD in general.

### Strengths

To our knowledge, this is the first study to present perioperative data in patients with IBD undergoing laparoscopic subtotal colectomy in relation to the use of epidural anesthesia. Furthermore, we provide detailed data concerning pain and opioid consumption in the early postoperative period. Finally, procedural data document the clinical challenges related to epidural anesthesia, including potential adverse events in the perioperative period.

### Limitations

The main limitation of our study is the observational design and the lack of randomization regarding epidural anesthesia. Hence, no standardization of the clinical indication for epidural anesthesia was present. Therefore, as seen in our data, placement of the epidural catheter was performed both pre- and postoperatively. For the latter, probably demonstrating an imminent pain issue (and possibly a surrogate marker of a more complicated postoperative course), and not as a preemptive analgesic strategy. Furthermore, it is possible that patients with an increased burden of comorbidity, and concomitant risk of complications, would receive epidural anesthesia more frequently during their surgical course, though our data cannot document this. Also, we chose only to register preoperative opioid use as a marker of chronic pain. It is possible that additional data concerning other types of analgesics would provide a more comprehensive picture of the burden of chronic pain in this patient group. Also, the intake of such analgesics may have impacted the clinical course of these patients. Another limitation is the fact that ERAS was not fully implemented in our department before 2020. Hence, patients before this time point, though treated with MIS, adhered to a conventional perioperative course protocol potentially impacting perioperative outcomes. Further, in our study, opioids were only administered by nurses, and not, e.g., by patient-controlled analgesia. There is a hypothetical risk that some patients would have received more opioids if they were able to control dosing of analgesics themselves, and not be dependent on a nurse not being preoccupied. A final limitation refers to the assessment of pain. In the PACU, NRS was employed; however, the number of assessments and timings were not standardized between patients. Also, assessments were only performed in the PACU. Further assessments made in the ward are needed to characterize pain in the entire postoperative period [40].

## Conclusions

This observational cohort study found a relatively low consumption of opioids and low pain scores in the early postoperative period following MIS subtotal colectomy. Epidural anesthesia was often associated with insufficient analgesic effects, and a substantial proportion of patients experienced adverse events related to epidural anesthesia. Epidural anesthesia remains a suitable analgesic treatment in selected patient groups, but our data do not support the use of epidural anesthesia as a standard in patients with IBD undergoing MIS subtotal colectomy.

## Data Availability

Data is available upon reasonable request.
